# Supplementary material for: The causal relationship between the human gut microbiota and pyogenic arthritis: a Mendelian randomization study
Source: Front Cell Infect Microbiol. 2024 Nov 26;14:1452480. doi: 10.3389/fcimb.2024.1452480 (PMC11629706; doi:10.3389/fcimb.2024.1452480)
Supplement: Supplementary file 1 [file DataSheet1.zip › mendelian test/ebi-a-GCST90027645.csv_leave_one_out.pdf]

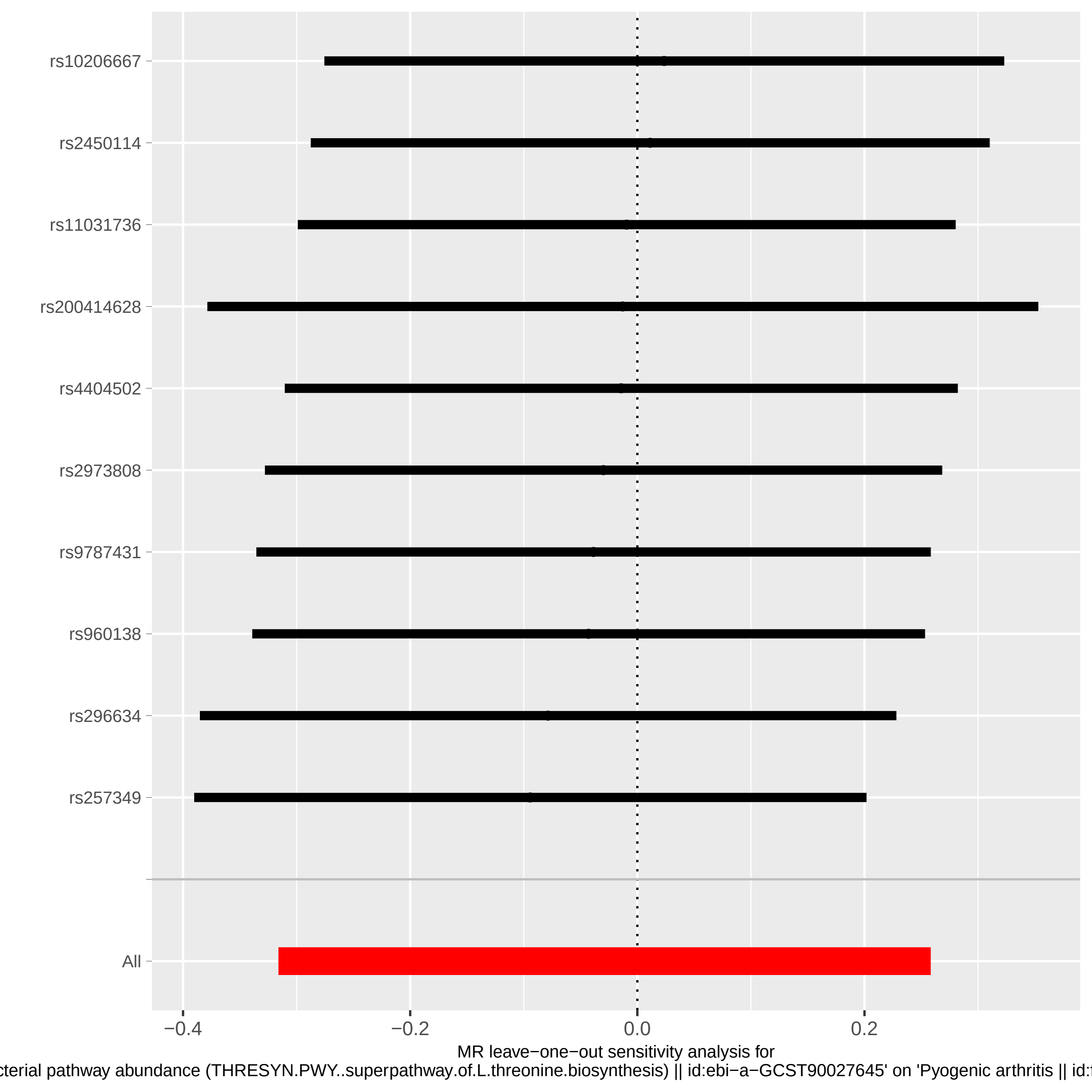

rs10206667
rs2450114
rs11031736
rs200414628
rs4404502
rs2973808
rs9787431
rs960138
rs296634
rs257349
All
−0.4
−0.2
0.0
0.2
MR leave−one−out sensitivity analysis for
cterial pathway abundance (THRESYN.PWY..superpathway.of.L.threonine.biosynthesis) || id:ebi−a−GCST90027645' on 'Pyogenic arthritis || id:
